# Alder, Nitrogen, and Lake Ecology: Terrestrial-Aquatic Linkages in the Postglacial History of Lone Spruce Pond, Southwestern Alaska

**DOI:** 10.1371/journal.pone.0169106

**Published:** 2017-01-11

**Authors:** Bianca B. Perren, Yarrow Axford, Darrell S. Kaufman

**Affiliations:** 1 British Antarctic Survey, High Cross, Cambridge, United Kingdom; 2 Dept of Earth and Planetary Sciences, Northwestern University, Evanston, Illinois, United States of America; 3 School of Earth Sciences & Environmental Sustainability, Northern Arizona University, Flagstaff, Arizona, United States of America; Universidade de Aveiro, PORTUGAL

## Abstract

Diatoms, combined with a multiproxy study of lake sediments (organic matter, N, δ^15^N, δ^13^C, biogenic silica, grain size, Cladocera and chironomids, *Alnus* pollen) from Lone Spruce Pond, Alaska detail the late-glacial to Holocene history of the lake and its response to regional climate and landscape change over the last 14.5 cal ka BP. We show that the immigration of alder (*Alnus viridis*) in the early Holocene marks the rise of available reactive nitrogen (Nr) in the lake as well as the establishment of a primarily planktonic diatom community. The later establishment of diatom *Discostella stelligera* is coupled to a rise of sedimentary δ^15^N, indicating diminished competition for this nutrient. This terrestrial-aquatic linkage demonstrates how profoundly vegetation may affect soil geochemistry, lake development, and lake ecology over millennial timescales. Furthermore, the response of the diatom community to strengthened stratification and N levels in the past confirms the sensitivity of planktonic diatom communities to changing thermal and nutrient regimes. These past ecosystem dynamics serve as an analogue for the nature of threshold-type ecological responses to current climate change and atmospheric nitrogen (Nr) deposition, but also for the larger changes we should anticipate under future climate, pollution, and vegetation succession scenarios in high-latitude and high-elevation regions.

## Introduction

Climate warming is expected to raise global temperatures by ~2°C by the end of this century, with even greater changes anticipated in the Arctic [1, 2]. At the same time, human alterations to the nitrogen cycle resulting from food and energy production (e.g. fossil fuel combustion) have increased reactive nitrogen (Nr) deposition to the Earth’s surface to as much as 10 kg N ha^-1^ yr^-1^ (less in remote areas, e.g. ~1 kg N ha^-1^ yr^-1^ in northern North America) [3]. Separately, ongoing atmospheric deposition of Nr and climate warming have the potential to strongly affect terrestrial, marine, and freshwater ecosystems (e.g. [2, 4–7]) but predicting the response of complex systems to these external stressors is a challenge as both occur simultaneously, perhaps synergistically, often with threshold-type effects.

In northern high latitude lakes, these stressors have an increased capacity for ecosystem disruption, as a) temperature changes are amplified in the Arctic [2, 8, 9] and b) despite their distance from industrial Nr sources, most boreal and arctic lakes are nutrient poor (oligotrophic) and their ecosystems are adapted to low nutrient levels, making them sensitive to even small changes in nutrient loading [10, 11]. Indeed, northern high latitude lake ecosystems have shown a dramatic response to climate warming in the last century [12, 13] and enhanced atmospheric Nr deposition has been shown to be occurring even in remote locations [14, 15] with attendant effects on ecosystems [6, 7, 16, 17].

In recent decades, the rise of small planktonic diatoms has become a feature of many Northern Hemisphere lakes (principally *Discostella stelligera* and similar small species of *Thalassiosiraceae*). This rise at the expense of larger planktonic or benthic forms has been linked with the onset of Anthropocene regime shifts, but attribution of the cause (i.e. climate warming, nutrient loading, or both) is still widely debated [18–22]. In many cases, stronger thermal stratification appears to be a common feature of these lakes [20, 22] but the role of allochthonous (atmospheric) nutrient additions have yet to be systematically ruled out, and in-lake bioassays suggest a significant response of these organisms to additional epilimnetic N in addition to climate-related parameters [23, 24].

Multiproxy paleolimnological methods allow for the exploration of past modes of natural variability and the way in which landscapes and climate have interacted with lake ecosystems and biogeochemical processes over century to millennial time scales. In this way, we can determine the varying roles of climate and nutrients on lake ecosystem dynamics in natural environments and offer some clues to decipher observational changes that are currently underway or are expected to take place in the future. This is especially true of lakes that contain significant *Discostella stelligera* populations over millennial timescales, and whose long-term interactions with landscape evolution and climate may shed light on its recent marked rise.

In southeastern Alaska, the linkages between postglacial development and landscape and lake evolution are fairly well constrained. Pioneering work [25, 26] has demonstrated that nitrogen fixers (e.g. *Dryas drummondii* and alder = *Alnus* spp.) play an enormous role in soil development in the early postglacial environment. Early postglacial landscapes are relatively poor in nutrients and rely on fixation of atmospheric N_2_ to augment soil and lake nutrient stores. While Nr can come from a number of sources (glacier outwash, cyanobacteria (e.g. *Nostoc* spp.), lichen, herb vegetation (e.g. *Dryas* spp.)), *Alnus* provides the greatest source of Nr to the local environment in Alaska (up to ~100 kg N ha^-1^ yr^-1^) through soil N pool development and subsequent leaching [27–29]. The role of *Alnus* in shaping nutrient regimes and evolution in lakes has been shown from a number of sites in southern Alaska [30, 31]. Work by Hu et al. [32] shows that these can have ecological effects: for example, the tandem rise of biogenic silica (BSi) and N during the Holocene at Grandfather Lake, Alaska, suggests that landscape colonization by alder stimulates lake productivity (primarily from BSi synthesizing diatoms). This relationship between N and planktonic diatoms has also been hinted at elsewhere in the low Arctic over the Holocene (e.g. Greenland) [33].

Here we present a diatom-based late-glacial and Holocene record from Lone Spruce Pond, southwestern Alaska that builds on previous work by Kaufman et al. [34], investigating the environmental history at the lake using a range of proxies (organic matter, C, N, BSi, δ^13^C, δ^15^N, grain size, Cladocera and chironomids, *Alnus* pollen). Using these proxies together, we show strong linkages between catchment and vegetation development, post-glacial climate, and the lake ecosystem (specifically the planktonic *Cyclotella s*.*l*. diatoms as well as invertebrate communities) over millennial timescales. Furthermore, we specifically explore the role of Nr in relation to alder establishment and the proliferation of planktonic diatom taxa (e.g. *Discostella stelligera*) and what they mean for ongoing lake ecosystem change in response to climate warming and nutrient loading in the Northern Hemisphere today.

## Study Area

**Fig 1:**
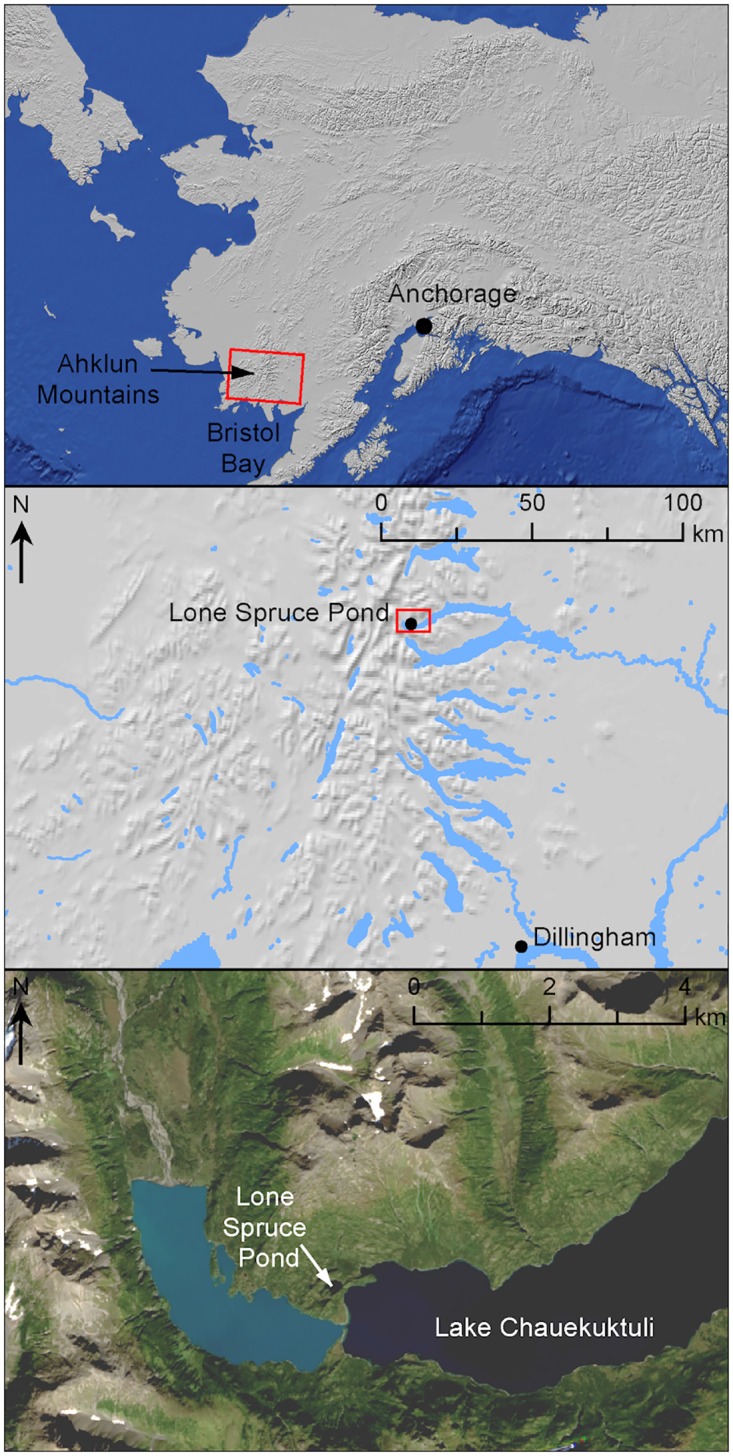
Map showing the location of Lone Spruce Pond, SW Alaska. The location of the pond within a) Alaska, b) the Ahklun Mountain Range, and c) adjacent to Lake Chauekuktuli (satellite imagery from USGS Earth Explorer).

Lone Spruce Pond (60.007°N, 159.143°W, 135 m asl) is located in the northeastern Ahklun Mountains in southwestern Alaska (Fig 1). The lake is small (0.05 km^2^) and relatively deep (maximum depth = 22 m) and has one outlet stream that drains into adjacent Lake Chuaekuktuli and precludes entry of anadromous fish due to a waterfall. The lake has a population of three-spined sticklebacks (*Gasterosteus aculeatus*). The lake catchment is small (0.14 km^2^) and currently unglaciated. It sits at the upper elevational limit of *Picea* and is densely vegetated with *Alnus viridis* as well as lesser quantities of *Betula* and *Salix* spp. The current climate at Lone Spruce Pond is between continental and maritime, with mean annual temperature of 1°C (between -9°C (January) and +13°C (July)), and 650 mm of precipitation, which peaks in late summer/early autumn [35]. The lake was glaciated during the Last Glacial Maximum [36], but modern glaciers are restricted to north-facing cirques at high elevation.

## Methods

We obtained a research permit from the US Fish and Wildlife Service, Togiak National Wildlife Refuge. Cores were taken from the deepest part of the lake and spliced together to form a composite 4.7 m long sequence using visible tephra stratigraphy (details in [34]). Diatoms were isolated from the sediments using standard oxidative laboratory procedures [37] and mounted on plain glass coverslips using Naphrax mountant. A known quantity of Divinyl benzene (DVB) microspheres was added to the finished slurries to estimate sedimentary diatom concentrations and fluxes [38]. At least 400 valves were identified and enumerated from each slide at 1000x under oil immersion light microscopy. Principal component analysis (PCA) was performed on centered, square root-transformed species assemblage data (consisting of >1% in any sample) using the program C2 [39]. Constrained cluster analysis (CONISS; [40]) using the broken-stick model [41] was performed on the species data using the R Rioja package [42]. Rate-of-change analysis of the diatom assemblage data was calculated by estimating the chord distance between each sample over the length of time elapsed [43].

Physical sedimentology and geochemical (MS, grain size, organic matter, N, δ^15^N, δ^13^C, BSi) and pollen, midge and Cladocera analyses and chronological methods are described in Kaufman et al. [34].

## Results

### Chronology

The age-depth model for the Lone Spruce Pond stratigraphy, based on ^14^C, tephra correlations, and Pu-based identification of nuclear weapons testing has been published previously and is only briefly summarized here. For full details see Kaufman et al. [34]. Sediment began accumulating in LSP ~ 25–21 cal ka BP. A possible disconformity and depositional hiatus occurs between 21–14.5 cal ka BP, during what is interpreted to be an unusually dry periglacial period in Alaskan prehistory. Continuous deposition is inferred since then.

### Diatoms

**Fig 2:**
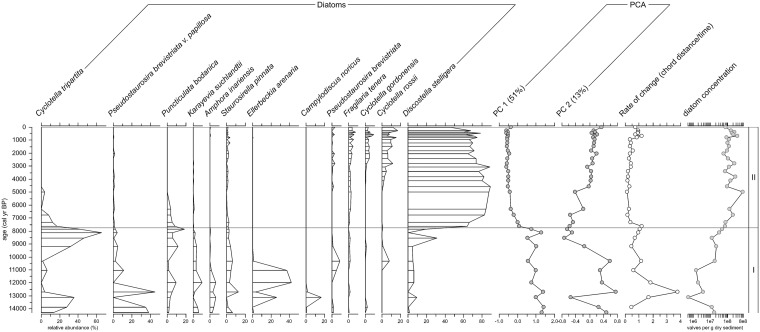
Diatom stratigraphy from Lone Spruce Pond. The figure shows the relative frequency of key diatom taxa, the first and second principal components (PC1 and PC2), the rate of change in the diatom community, and the concentration of diatoms in the sediments.

A total of 109 species were identified from the core material. Of these, 56 occurred in relative abundances >1% in any one sample. The most common and abundant taxa, *Discostella stelligera*, *Cyclotella tripartita (= Lindavia tripartita)*, *Cyclotella rossii (= L*. *Rossii)*, *Puncticulata bodanica (= L*. *bodanica)*, *Pseudostaurosira brevistriata v*. *papillosa*, *and Staurosirella pinnata*, are typical of dimictic, oligotrophic lakes and are found throughout the circumpolar north (Fig 2).

Constrained cluster analysis using the broken stick model delineated two significant zones in the Lone Spruce Pond record which are also reflected in the diatom PCA scores: one before 7.8 cal ka BP and one after. Diatoms were rare or absent in the lowermost sediments (390–450 cm) corresponding to the LGM (>20 cal ka BP). Diatoms appeared around 14.3 cal ka BP but remained at relatively low concentrations until ~7.6 cal ka BP. These zone 1, earliest sedimentary assemblages, are dominated by benthic *Fragilariaciae*, specifically *Pseudostaurosira brevistriata v*. *papillosa* and *Staurosirella pinnata*, as well as by planktonic *Cyclotella tripartita*, suggesting relatively clear, oligotrophic lake conditions (cf. Perren et al., 2012b). Periods of extremely low diatom abundance, where counts were below the standard 400 valves (between 13–11 cal ka BP) were characterized by high relative frequencies of *Pseudostaurosira brevistriata v*. *papillosa*, *Ellerbeckia arenaria* and *Campylodiscus hibernicus*. The latter two taxa are found in shallow water minerogenic environments [44], while *C*. *hibernicus* has also been found in sediments of deep silt-dominated lakes with strong ice cover regimes elsewhere in the High Arctic [45, 46]. A renewed peak of medium-sized planktonic *Cyclotella tripartita* occurs at 8.2 cal ka BP, followed by a small peak in *Puncticulata bodanica*, whereupon the present small planktonic *Discostella stelligera*-dominated community (zone 2) was established. The present planktonic community has been remarkably stable since ~6 cal ka BP, although it became increasingly diverse over the late Holocene and Neoglacial, containing higher relative contributions of planktonic taxa *Cyclotella rossii*, *C*. *gordonensis*, and *Fragilaria tenera* towards the present.

Rate-of-change analysis of the diatom assemblage data shows three salient features: 1) the largest peak at the onset of the Younger Dryas (12.9–11.7 cal ka BP) transition suggesting widespread floristic change associated with this cold period, followed by 2) continued ecological instability until the beginning of diatom zone 2 (~8 cal ka BP) whereupon the lake becomes more stable, and 3) an increase in species turnover/instability in the most recent (post 1300 CE period) coinciding broadly with the Little Ice Age (LIA; ~1300–1900 CE).

### Geochemistry

**Fig 3:**
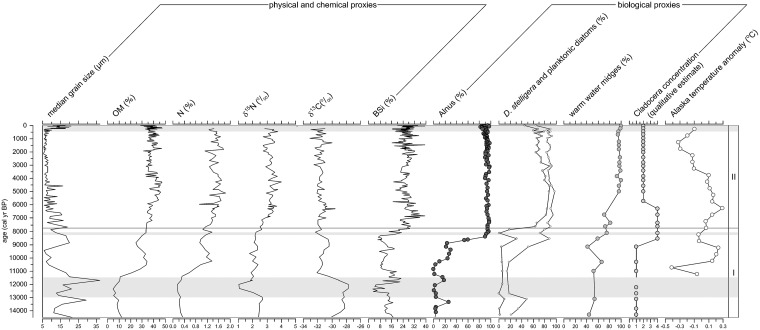
Key geochemical, biological, and diatom-related data from Lone Spruce Pond. Alaska temperature anomaly from Kaufman et al. ([47]; their figure 14f), other proxies from [34].

Geochemical results are discussed in detail in Kaufman et al. [34] and are only briefly summarized here for comparative purposes. Percentages of organic matter (OM), nitrogen (N) and biogenic silica (BSi) each has their lowest values in the earliest part of the record, with minima during the Younger Dryas (Fig 3). BSi and δ^15^N have marked minima at its onset. After the Younger Dryas, OM, N, and δ^15^N values rise and reach maxima between 6.5–4 cal ka BP, whereas the BSi peak occurs earlier around 7 ka BP. These geochemical trends are the opposite of the grain size and δ^13^C, which show maximum values at the beginning of the record at the glacial-interglacial transition, and especially bracketing the Younger Dryas. Lowest values of grain size and δ^13^C prevail after 8 cal ka BP, but median grain size increases again during the LIA.

### Midges and Cladocera

Invertebrate assemblages (Chironomidae, Chaoboridae and Cladocera) are described in Kaufman et al. [34]. Major shifts in the invertebrate assemblages occur: (1) after the Younger Dryas, at the onset of the Holocene, with high percentages of *Micropsectra* and a rise in *Sergentia* and a brief occuurence of Chaoboridae; (2) during the centuries just prior to 8 cal ka BP, with the establishment of *Corynocera ambigua* and *Limophyes* and decline in *Micropsectra* and Cladocera peak at this time; and (3) during the mid-Holocene around ~6 cal ka BP, when *Micropsectra* declines precipitously and *Sergentia* and *Corynocera ambigua* become established as the dominant taxa through the mid- and late- Holocene until the present.

## Discussion

Diatoms combined with other biological and geochemical proxies from Lone Spruce Pond detail the limnological response to changing climate and landscape evolution during the late-glacial-interglacial transition and Holocene epoch. These can broadly be divided into two categories: before *Alnus* (>14.5 ka BP- 8 ka BP), and after *Alnus* (post-8 ka BP).

### Before *Alnus*: >14.5 ka– 8 ka BP

In the Late Glacial sediments older than 14.5 cal ka BP, few diatoms are present in the sediments at Lone Spruce Pond. Low BSi and OM suggest that in-lake production and terrestrial production were both very low, and high mineral matter content suggest that Lone Spruce Pond was an unproductive, turbid, and ice-covered proglacial lake. Chironomid, *Chaoborus*, and Cladocera remains are absent in the early part of this period. Towards the end of the glacial period, all three of these invertebrate groups appear, indicating warmer summer temperatures, and pollen records attest to the local establishment of sparse shrubby vegetation [34]. However, the lack of diatom remains suggests that the light climate was still poorly suited to primary production as is the case with many proglacial lakes today.

The beginning of the Bølling warm period (~14.5 cal ka BP) marks decoupling from the glacial meltwater supply and the beginning of organic, non-glacial sedimentation and the onset of diatom production in Lone Spruce Pond. Diatom concentrations are low, compared with later in the record, as are BSi and OM. δ^13^C values are high, which suggests that OM was dominated by in-lake production rather than a terrestrially-derived carbon source. Midge assemblages during this period suggest July temperatures of at least 10.7°C [34]. Low diatom abundance dominated by planktonic *Cyclotella tripartita* as well as *Pseudostaurosira brevistriata v*. *papillosa*, and *Staurosirella pinnata* suggests that the lake was still somewhat turbid, with weak and/or deep summer stratification [21], high pH, and low nutrient availability. Although this period marks the emergence of the diatom flora in Lone Spruce Pond, the aquatic paleoecology suggests that it is still an ice-proximal, early postglacial environment, with little slope stability offered by terrestrial vegetation, low attendant nutrient concentrations, and a relatively compromised light environment.

The environmental response to the Younger Dryas is varied across Alaska, with some regions showing cold, arid conditions, and others showing enhanced moisture [48–50]. The onset of the Younger Dryas in Lone Spruce Pond interrupts the progressive development of the landscape with a fundamental reversal of the ecology of the lake, as shown by the diatom-based rate-of-change analysis (Fig 2). BSi drops dramatically, OM and N values are low, grain size increases, δ^15^N drops to near-air values (0‰), chironomid abundance is low [34], and diatoms are infrequent. The large, heavily silicified, benthic forms that occupy the lake at this period (*Campylodiscus hibernicus* and *Ellerbeckia arenaria*) suggest a lowered lake level, a greater influx of minerogenic matter, and a very poor light quality. These aquatic conditions suggest that climate was cold and arid at this time and may have limited diatom production, and destabilized the catchment.

A warmer, moister climate prevailed at the end of the Younger Dryas allowing for the expansion of alder shrubs into the catchment and for the concomitant increase in C and N from terrestrial sources. From values near 0% at the end of the Younger Dryas, *Alnus* values rapidly rose to 90% of the pollen relative abundance circa ~8.5 cal ka BP at Lone Spruce Pond, documenting the rapid immigration of alder into the catchment. Prior to *Alnus* immigration, BSi values are low, diatom concentrations are low, and high proportions of sand suggest an unstable catchment and reduced water clarity. However, both chironomid concentration and diversity rose and *Chaoborus* flourished briefly (for about 1 ka), suggesting, that despite an unstable catchment, warm climatic conditions prevailed [34]. After the establishment of *Alnus*, OM and N values increase as soil nutrient pools develop, and inorganic sedimentation decreases. Diatoms start at low levels at 8.5 cal ka BP, likely as a result of still nascent nutrient stores, but then shift to a predominantly oligotrophic phytoplankton-dominated community. Diatom assemblage rates of change are high, likely reflecting the transitory nature of this phase of lake development. Chironomid assemblages also changed at 8.5 cal ka BP with the rise of *Alnus*, most notably with an increase in *Limnophyes* and *Corynocera ambigua*, and the appearance of *Tanytarsus chinyensis*-type at the expense of *Micropsectra*.

### Post-*Alnus*: 8–0 cal ka BP

With the rise and establishment of *Alnus* c. 8 cal ka BP, biological and chemical proxies appear to follow two response pathways: they either shift abruptly and then exhibit stable values throughout the rest of the Holocene (e.g. BSi, *Alnus*, total planktonic diatoms) or shift after the marked *Alnus* rise (by ~1–2 ka; e.g. OM, N, δ^15^N, chironomids, *Discostella stelligera*) to a more stable state. *Cladocera* flourish abruptly with the establishment of *Alnus*, but only remain at very high concentrations until shortly before 6 cal ka BP. Sometime between 6.5 and 5 cal ka BP and at least roughly coincident with the mid-Holocene drop in *Cladocera*, chironomid assemblages also shift abruptly: *Micropsectra* (an indicator of oligotrophic, well-oxygenated, well-mixed environments; [51]) declines dramatically and *Corynocera*. *ambigua* (which is associated with shallow lakes in a regional training set; [52]) and especially oxy-regulatory *Sergentia* become more abundant, suggesting increased trophic status and shallower summer stratification. Median grain size values decline through several millennia following the final establishment of *Alnus* and reach stable, lowest values between 4 and ~0.7 cal ka BP. This period ~7.5–4 cal ka BP appears to have been the most conducive to primary production at Lone Spruce Pond, with stable catchment vegetation and soils, abundant nutrients, and a stronger stratification regime that allowed for the local proliferation of planktonic diatoms, especially *D*. *stelligera*.

There is no clear shift to the Neoglacial (post-4 cal ka BP) in this record, but the diversification of planktonic flora in the last several thousand years (increasing quantities of *Fragilaria tenera*, *D*. *gordonensis* and *C*. *rossii* since 3–4 cal ka BP) suggest some change in thermal/nutrient dynamics. Kaufman et al. [34] suggest a shift to cooler Neoglacial conditions beginning 2.4 cal ka BP. This mid- to late-Holocene stability is only broken during the Little Ice Age (starting ~1300 CE) when planktonic diatoms decrease and median grain size increases.

### *Alnus*, Nr, and *Cyclotella sensu lato*

**Fig 4:**
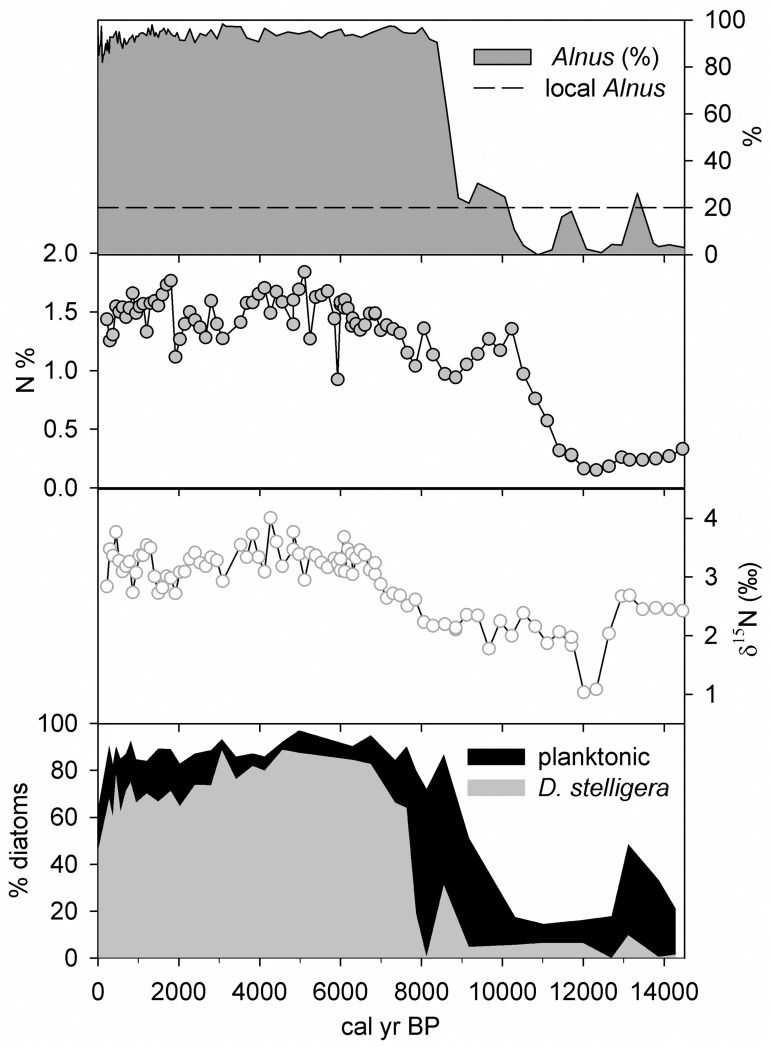
Summary diagram showing the linkages between *Alnus* pollen, N, δ^15^N, and planktonic diatoms in Lone Spruce Pond. Note: small cyclotelloid diatom *Discotella stelligera* in grey. Pollen and geochemical data are from Kaufman et al. [34]

An important ecological transition occurs in the Lone Spruce Pond record at c. 8 cal kyr BP when *Alnus* pollen reaches ~90%, comparable to core-top levels and presumably marking when dense alder thickets surrounding the lake became established. It is also at this point that planktonic diatoms reach ~80% of the total diatom flora. The relationship between alder and planktonic diatoms is shown in Fig 4. Here, *Alnus* immigration appears to have served two functions: 1) landscape stabilization and concomitant light amelioration (with a possible reduction in wind stress), and 2) the provision of a large source of Nr, both of which are critical for phytoplankton growth. Initial diatoms at this early stage of development are larger forms (e.g. *Puncticulata bodanica*, *Cyclotella tripartita/comensis*), which favour deeper mixing regimes (weak stratification) and tolerate lower nutrient levels [21, 23].

Slightly after the initial *Alnus* and phytoplankton rise, δ^15^N, diatom concentration and *Discostella stelligera* reach their respective maxima, between ~6.5–4 cal ka BP, corresponding also with the highest sediment N_tot_ concentrations and warmth inferred by other biological proxies at Lone Spruce Pond (e.g. invertebrates; Kaufman et al., 2012) and regionally [34]. Invertebrate assemblages mirror these changes with a shift towards oxy-regulatory and higher nutrient-associated taxa [34]. Warmer summers would have not only changed the thermal structure (and reduced the relative mixing depth) but also increased the soil nutrient and dissolved organic carbon (DOC) pool resulting in a greater supply of N to the lake, and shallower stratification regimes from reduced transparency [53] (Fee et al., 1996). These factors favour *D*. *stelligera* growth [21]. Enhanced N cycling from phytoplankton growth also increases δ^15^N values. Amongst other factors, reduction of competition as the Nr pool increases would also lead to higher δ^15^N. The clear relationship between diatom concentration and δ^15^N values (Fig 4) suggests the increasing phytoplankton population as a plausible driver of the rising δ^15^N values.

### Diatom phytoplankton and climate

As in other boreal and arctic regions, the relationship between diatoms and paleoclimate at Lone Spruce Pond is not straightforward [33, 54]. At many sites in the Northern Hemisphere, climate records suggest that maximum Holocene warmth occurred with the high summer insolation values following the Younger Dryas (~11–9 cal ka BP; [55]). However, in Alaska, reconstructions show a high degree of spatial variability during the early Holocene; and most paleoclimate reconstructions show maximum warmth between 7 and 5 cal ka BP [56]. This is also true of Lone Spruce Pond, where biological and geochemical proxies appear to record a mid-Holocene thermal maximum (~7–4 cal ka BP).

Although the presence of alder seems to exert a first-order control on phytoplankton composition in Lone Spruce Pond, via its effect on local N cycling, climate has both direct and indirect effects on the ecosystem behavior as well. The three major climate perturbations of the last 14.5 ka are clearly recorded in the phytoplankton at LSP: the Younger Dryas, a cooling at 8.2 cal ka BP, and the Little Ice Age. Reductions in diatom phytoplankton percentages accompany cooler periods (Fig 3), which would have reduced the strength of summer stratification, resulting in a less favourable light climate and nutrient regime for planktonic diatoms, especially *D*. *stelligera*. Grain size is also coarser during these cooler periods, which suggest either remobilization of the catchment or nearby aeolian deposits and the subsequent reduction of light environment for planktonic communities. Indirectly, climate warming also drives changes in the biogeochemical cycling which can affect lake ecology. For example, higher concentrations of epilimnetic chlorophyll and DOC, resulting from nutrient enrichment and enhanced soil development, limit the thermocline depth [53], promoting the establishment of small planktonic taxa [23] and reinforcing the effects of climate warming on the lake ecosystem.

### Implications for the anthropocene

The Anthropocene has seen not only the rise of temperatures globally, but also an acceleration of atmospheric- and land- derived pollutants, land use pressures, and invasive species, amongst others, all of which act as multiple stressors on aquatic ecosystems. One way of assessing ecosystem responses to these stressors is to look at previous climate periods and their temperature and nutrient regimes as potential analogues. At Lone Spruce Pond, the rise of temperatures and nutrients in the early to mid-Holocene provides an excellent analogue for the limnological direction of current climate change and atmospheric Nr deposition, as well as the resulting threshold ecological response. At Lone Spruce Pond, *Discostella stelligera* rises dramatically at the onset of the nitrogen-rich *Alnus* period. The immigration of *Alnus* likely stabilized the catchment, also ameliorating the light climate, but the rise and dominance of *Discostella stelligera* appears to be directly related to the surge of available nitrogen in the system. If this is indeed the case, and *D*. *stelligera* thrives not only in warmer periods of enhanced thermal stratification, but responds dramatically to increased nutrient loading (see also [21, 24]) then we should expect these threshold-type responses at the expense of larger planktonic and benthic forms in oligotrophic lakes in the decades to come. Furthermore, as treeline expands northward and into higher elevations with continued climate warming, we should expect large changes in lake ecosystems resulting not only from climate change, but from added complexities resulting from vegetation and catchment development. This is true where *Alnus* spp. ranges are expected to expand, but also true of the broader boreal forest, taiga, and tundra regions, where changing vegetation from either migrating treeline or shrubification can result in large changes in landscape biogeochemistry, which has cascading effects on ecosystems.

## Conclusions

Planktonic diatoms from Lone Spruce Pond, Alaska provide important insights into the linkages between catchment vegetation, climate, geochemistry and lake ecology over millennial timescales. The role of *Alnus* and attendant N-fixation is fundamental to the postglacial development of the lake by driving new limnological regimes, especially for the planktonic diatom flora, which form a critical part of aquatic food webs. The early- to mid-Holocene rise of planktonic *Discostella stelligera* in Lone Spruce Pond mirrors those regime shifts currently affecting northern hemisphere lakes and suggests that rising N levels under warm climate conditions must be considered as a factor in their success over other planktonic taxa in northern oligotrophic lakes today. Finally, multiproxy analyses of late-glacial and Holocene lake sediments can tell us not only about past ecological changes and the linkages between climates, catchments, and lake ecology, but also about threshold-type responses in natural ecosystem dynamics that are a critical and poorly understood component of ongoing and future Anthropocene ecological change.
